# Structural basis of blocking integrin activation and deactivation for anti-inflammation

**DOI:** 10.1186/s12929-015-0159-6

**Published:** 2015-07-08

**Authors:** Eun Jeong Park, Yoshikazu Yuki, Hiroshi Kiyono, Motomu Shimaoka

**Affiliations:** Department of Molecular Pathobiology and Cell Adhesion Biology, Mie University Graduate School of Medicine, Mie, 514-8507 Japan; Division of Mucosal Immunology, Department of Microbiology and Immunology, The Institute of Medical Science, The University of Tokyo, Tokyo, 108-8639 Japan; International Research and Development Center for Mucosal Vaccine, The Institute of Medical Science, The University of Tokyo, Tokyo, 108-8639 Japan

**Keywords:** Integrin, Affinity, Activation, Deactivation, Leukocyte, Inflammation, Talin, Kindlin

## Abstract

Integrins mediate leukocyte accumulation to the sites of inflammation, thereby enhancing their potential as an important therapeutic target for inflammatory disorders. Integrin activation triggered by inflammatory mediators or signaling pathway is a key step to initiate leukocyte migration to inflamed tissues; however, an appropriately regulated integrin deactivation is indispensable for maintaining productive leukocyte migration. While typical integrin antagonists that block integrin activation target the initiation of leukocyte migration, a novel class of experimental compounds has been designed to block integrin deactivation, thereby perturbing the progression of cell migration. Current review discusses the mechanisms by which integrin is activated and subsequently deactivated by focusing on its structure-function relationship.

## Introduction

Integrins are the largest family of cell adhesion molecules that mediate cell-to-cell and cell-to-matrix interactions in a broad range of biological phenomenon such as host defense, hemostasis, wound healing, angiogenesis, organ development [3, 29, 43, 63, 73]. These functions are achieved via integrin bidirectional signaling across plasma membrane [34, 55]. Inside-out signaling takes place upon association of intracellular activators (*e.g.*, talin, kindlins) with integrin cytoplasmic domains, leading to transition of integrin conformation to high affinity for binding ligands. Upon ligand binding, integrins undergo clustering and transmit their outside-in signals to the cytoplasmic domains, leading to forming focal adhesions that connect to actin filaments for many cellular processes. While integrins are expressed virtually in all cell types, a subset of integrins including αLβ2, αMβ2, α4β1, and α4β7 are predominantly expressed on leukocytes, thereby regulating immune cell trafficking to lymphoid tissues and sites of inflammation.

Integrins play a critical role in the regulation of extravasation of leukocytes to sites of inflammation [5, 11, 12]. During rolling along the endothelial cells via selectins, leukocytes encounter chemokines expressed on endothelial cells [87]. The integrin activation by chemokine enables abrupt arrest of cells on endothelial integrin ligands. Subsequently adherent leukocytes leave the initial point of arrest, and, thereby, crawl along the endothelial apical surface to a so-called hot spot where they undergo transmigration across the endothelial cells [69]. Crawling and transendothelial migration (TEM) requires a dynamic balance of up- and down-regulation of cellular adhesiveness that is achieved by not only integrin activation but also properly regulated integrin deactivation [52]. Here we review the molecular mechanisms that regulate integrin activation and deactivation.

## Review

### Leukocyte interaction with endothelial cells to enter inflamed tissues

Leukocyte interaction with endothelial cells represents early events during inflammation or immune surveillance and occurs through selectin-mediated rolling, chemokine-driven activation, and integrin-dependent arrest [37, 44, 82]. Binding of chemokines to G protein-coupled receptors (GPCRs) triggers rapid arrest of rolling leukocytes in which leukocyte integrins (*e.g.*, αLβ2 or α4β1) are activated to adhere to the ligands such as intercellular adhesion molecule 1 (ICAM1) or vascular cell-adhesion molecule 1 (VCAM1) on endothelial cells [65]. Leukocytes undergo intravascular crawling via αMβ2-ICAM1 interaction until finding the “hot spots” proper for emigrating into inflamed tissues [53]. Leukocyte integrins, αLβ2 and α4β1, interact with junctional adhesion molecules (JAMs) such as JAM-A and JAM-B, respectively, on endothelial cells to facilitate TEM of the leukocytes as a final step in the homing cascade to inflamed tissues [40]. Formation of an endothelial docking structure with actin-based membrane protrusions is thought to raise efficiency of leukocyte TEM during inflammation [10, 45].

### Structure and conformational regulation of integrins

**Fig. 1:**
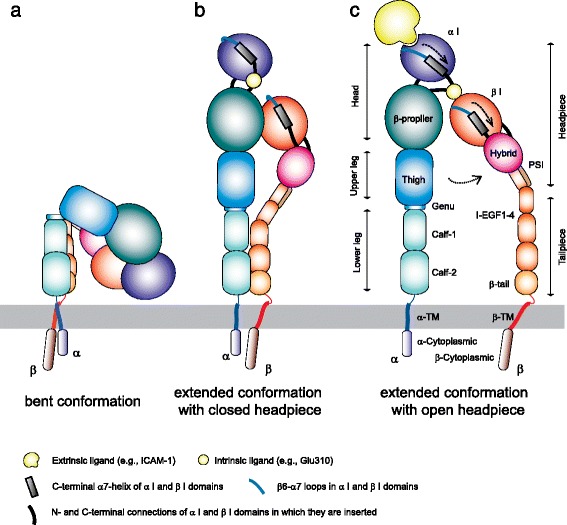
Different integrin conformations. **a** Bent conformation containing the closed headpiece (with low-affinity I domain). **b** Extended conformation containing the closed headpiece (with intermediate-affinity I-domain). **c** Extended conformation containing the open headpiece (with the high-affinity I domain). I-EGF, integrin-epidermal growth factor; PSI, plexin/semaphorin/integrin; TM, transmembrane

Integrins are α/β heterodimeric membrane proteins that exhibit a characteristic feature of complex multi-domain organization [76, 77]. Integrins on resting cells are maintained in a default inactive state, in which the headpiece is folded back to the leg pieces [8], thereby exhibiting a bent conformation (Fig. 1a). Of note, the cytoplasmic parts of integrin α and β subunits are associated, thereby stabilizing the bent conformation [80]. In this inactive bent conformation, the ligand binding domain is not only oriented unfavorable for interacting with ligand on the opposing cells, but also in the low affinity configuration. Upon integrin activation, the interface between headpiece and tailpiece is opened in a switchblade-like movement, thereby exhibiting an extended conformation, in which the ligand binding headpiece is oriented favorable toward the ligand on the opposing cells [7, 74]. The conversion from the inactive bent conformation to the active extended conformation with open headpiece is triggered by the separation of the α/β cytoplasmic domains [34, 79], and is linked to the structural rearrangements in the ligand binding domain that leads to the high-affinity configuration (Fig. 1c). Along the course of activation dependent conformation conversion from the bent conformation to the extended form with open headpiece, an extended form with closed headpiece has been proposed as an intermediate state that possess intermediate affinity to the ligand (Fig. 1b) [7, 74].

Half of the integrin α subunits and all of β subunits possess a von Willebrand factor-type A domain, which is also known as an inserted (I) domain [26, 67]. The α I and β I domains adopt a Rossmann fold that contains a metal ion-dependent adhesion site (MIDAS) located on the top, whereas its C- and N-terminal connections located on the distal bottom face [25, 36, 67, 89]. The ability of the I domain to bind ligand is regulated by conformational changes. The affinity of the I domain for its ligand is dramatically enhanced by a “piston-like” downward axial displacement of its C-terminal helix. The C-terminal downward shift is conformationally linked to the conversion of the MIDAS to the high-affinity open configuration [28, 66, 68, 83]. The C-terminal α helix contains an invariant isoleucine residue at the bottom. The side chain of the invariant isoleucine is deeply embedded to the hydrophobic pocket underneath the helix, thereby acting as a ratchet that prevents the helix from moving down easily. The invariant isoleucine serves as an important intrinsic structural component to maintain a default low-affinity I domain, thereby constituting the mechanisms of integrin deactivation.

The association of the α and β integrin cytoplasmic tails functions as a clasp that stabilizes the low-affinity bent conformation. The arginine residue in the GFFKR motif makes a salt bridge with the conserved acidic residue (aspartate or glutamate) at the membrane proximal region of the β cytoplasmic domain. The cytoplasmic salt bridge plays a critical role in “clasping” the α/β cytoplasmic domains together, thereby serving as another intrinsic structural component to maintain a default low-affinity integrin conformation [24, 38, 39]. The activation of chemokine receptors initiates an intracellular signaling cascade that eventually impinges upon the integrin cytoplasmic tails. Binding to the integrin cytoplasmic domains of adaptor molecules triggers a dissociation of the integrin cytoplasmic tails, thereby triggering integrin activation of the β I domain [9, 22, 85]. Activated β I domain binds to an intrinsic ligand (a conserved acidic residue) at the linker region connecting to the C-terminal helix of the α I domain [62]. This inter-domain interaction (*i.e.*, binding of the β I domain to the intrinsic ligand) facilitates the pulling down the C-terminal helix of the α I domain, thereby inducing the high-affinity open MIDAS conformation of the α I domain that is competent for the external ligand.

### Fine-tuning integrin activation and deactivation

Cell migration requires cycles of integrin activation and deactivation [95]. A simplified view is that at the front of migrating cells integrin activation takes place, thereby mediating cell adhesion, while at the rear of those cells integrin deactivation occurs, thereby facilitating cell de-adhesion [47]. Several mechanisms are involved in the regulation of the balance between activation and deactivation, thereby fine-tuning integrin-mediated cell adhesion and migration.

**Fig. 2:**
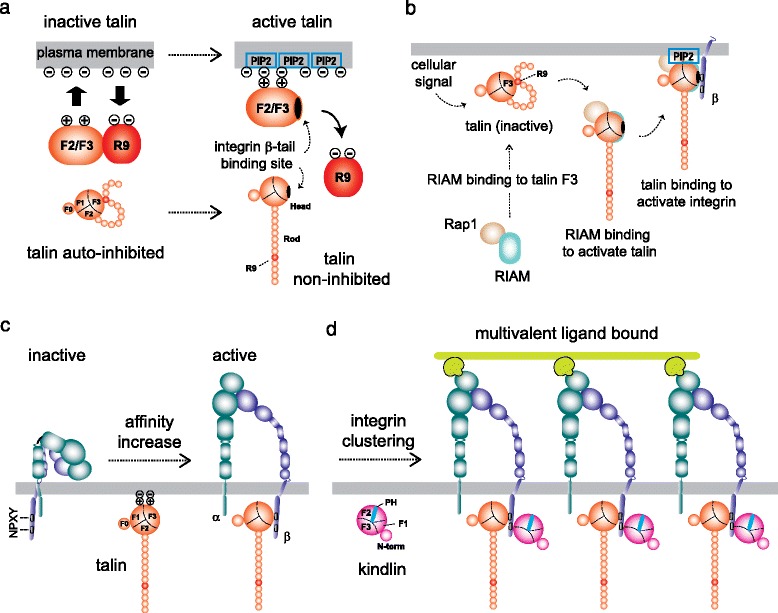
Integrin activator talin and co-activator kindlin. **a** The auto-inhibition of talin is release either by phosphorylation or partial proteolysis. **b** Recruitment of talin to integrin is mediated by binding Rap1-RIAM complex. **c** Binding of talin to the integrin triggers the dissociation of the α/β integrin cytoplasmic domains, thereby inducing active extended conformation. **d** Kindlin acts as a co-activator to stabilize integrin-mediated cell adhesion that involves multivalent ligand binding

Talin is the cytoplasmic adaptor molecule that plays the central role in triggering integrin activation. Talin is a large, actin-binding, cytoskeletal protein that comprises an N-terminal globular talin head that is connected, via a long unstructured linker, to a C-terminal long tail-like talin rod. The talin head contains 4 FERM domains (F0, F1, F2, and F3) [19, 49]. The talin head F3 domain contains a primary integrin binding site that directly interact with the integrin β cytoplasmic tail. Talin head also binds to the inner surface of the plasma membrane, which is mediated by electrostatic attraction of a cluster of basic residues in talin head domains with acidic plasma-membrane phospholipids such as phosphatidylinositol 4,5-bisphosphate (PIP2). To activate integrin only on demand, talin binding to integrin needs to be regulated. In an inactive state, talin adopts the auto-inhibition conformation (Fig. 2a) [13, 18]. The auto-inhibition conformation in talin is released by the presence of PIP2 [84]. This could be made possible through recruitment of talin to PIP2-rich membrane microenvironments that also contain integrins. This pull-push model for talin activation is shown in Fig. 2a. As an alternative to PIP2, some proteases in the cytoplasm such as calpain or metalloproteinase-2 possess a limited proteolysis effect to unmask the auto-inhibition conformation of talin potentially liberating the talin head available for integrin activation [72, 90]. Also, Rap1-RIAM complex plays an important role in chemokine- and TCR-triggered up-regulation of integrin activity in leukocytes. When talin is recruited to the plasma membrane upon cellular signal, RIAM in the Rap1-RIAM complex binds to the talin [91]. This binding activates talin to associate with β integrin cytoplasmic tail, which mediates integrin activation (Fig. 2b). The talin F3 domain primarily binds the membrane-proximal NPXY motif of the integrin β tail, and then forms a non-covalent interaction with a conserved acidic residue at the membrane proximal region of the integrin β tail [4]. The salt bridge helps to stabilize the talin-integrin interaction as well as unclasp the salt bridge formed between the α and β integrin cytoplasmic tails (Fig. 2c). Thus, talin appears to utilize the plasma membrane binding as a pivot point to exert a robust effect on the integrin cytoplasmic tail. In this way, talin causes the separation of the integrin α and β cytoplasmic tails, thereby triggering integrin activation.

Kindlins (kindlin 1, kindlin 2, and kindlin 3) are another family of FERM domain-containing proteins that are predicted to adopt a similar structure to the talin head. A hematopoietic cell-specific kindlin 3 has been found as an integrin co-activator that cooperates with talin. Kindlin 3 is required for effector T cells in α4β1 and αLβ2 integrin binding to and stabilization of the interaction with ligand, especially at low level of integrin ligand [46]. Kindlin binds to the membrane distal NPXY motif of the β cytoplasmic domain, as opposed to talin that binds to the membrane-proximal NPXY motif [93]. Kindlin does not compete with talin for integrin and augments multivalent ligand binding capacity by promoting the clustering talin-activated integrins (Fig. 2d) [94]. In contrast to talin, kindlin by itself is unable to induce the separation of the integrin cytoplasmic association. Kindlin rather functions as a co-activator to talin, thereby potentiating talin-mediated integrin activation and cell adhesion [94].

Kindlin 1 is overexpressed in lung and colon carcinomas and hence targeting kindlin 1 can be effective to restrain metastasis in some cancers [71, 86]. Loss-of-function mutations in kindlin1 caused Kindler syndrome (KS) in which the keratinocytes from KS patients had a defect in motility due to impaired activation of β1 integrin [23, 35, 70]. Kindlin 2 itself appears to involve in suppressing cancer cell migration [21, 57], although little has been reported for direct evidence on human disease related to the genetic defect in the kindlin 2. An invasive breast cancer cell line (TMX2-28) highly expressed kindlin 2 that played a critical role in cell invasion, since knocking down of kindlin 2 repressed cell invasiveness [20]. Mutations in kindlin 3 cause leukocyte adhesion deficiency (LAD) type-III, a primary immune deficiency that manifests unresponsiveness of agonist-triggered integrin-mediated leukocyte adhesion and platelet aggregation [14, 42, 60, 75]. The kindlin 3 knockout mice exhibited the LAD-III like phenotypes including perturbed integrin-mediated adhesion of leukocytes to endothelial cells [48].

### Suppression of integrin activation by interfering with talin binding

This section describes cytoplasmic molecules that have been shown to interfere with talin binding to integrins, thereby stabilizing the inactive bent conformation. Phosphotyrosine-binding (PTB) domain-containing proteins, Dok1 (docking protein 1) and ICAP1 (integrin cytoplasmic domain-associated protein 1), have been shown, like filamin, to compete with talin for binding to the integrin cytoplasmic domain. Dok1 has been shown to bind to the NPXY motif [50]. However, only talin but not Dok1 stabilizes the open active conformation of integrin [85]. The inability to disrupt the clasping salt-bridge makes Dok1 unable to induce the separation of the integrin α/β cytoplasmic tails needed for activation. The mode by which Dok1 competes with talin is regulated by integrin tyrosine phosphorylation. Binding of talin, but not of Dok1, in integrin β tails is reduced by integrin tyrosine phosphorylation [1]. Thus phosphorylation makes integrin inactive.

SHARPIN (SHANK-associated RH domain-interacting protein) gene mutations naturally occurring in mice were found to cause chronic proliferative dermatitis, a systemic inflammation involving multiple organs [64]. More recently, SHARPIN has been found as an inhibitor of integrin activation that interacts with the α integrin cytoplasmic and, thereby, interferes with talin binding to integrin and subsequent integrin activation [56]. SHARPIN binds to the α integrin membrane proximal region that contains the conserved GFFKR motif to form the clasping salt-bridge with the β subunit [56]. SHARPIN binding does not involve the arginine residue in the GFFKR, thereby maintaining the formation of the clasping salt-bridge [56]. Furthermore, SHARPIN binding to the α integrin tail inhibits talin and kindlin binding to the β integrin tail presumably through steric hindrance [56]. SHARPIN deficiency has been shown to enhance integrin-mediated cell adhesion and reduce its migration velocity [54]. In addition to its role of inactivating integrins, SHARPIN also functions as an ubiquitin binding protein that plays an important role in the regulation of NF-κB signaling [30]. Thus, chronic inflammation occurred in SHARPIN mutant mice could be due to both aberrant integrin activation and NF-κB signaling.

### Integrin intrinsic components to stabilize the default inactive conformation

#### Cytoplasmic GFFKR motif

**Fig. 3:**
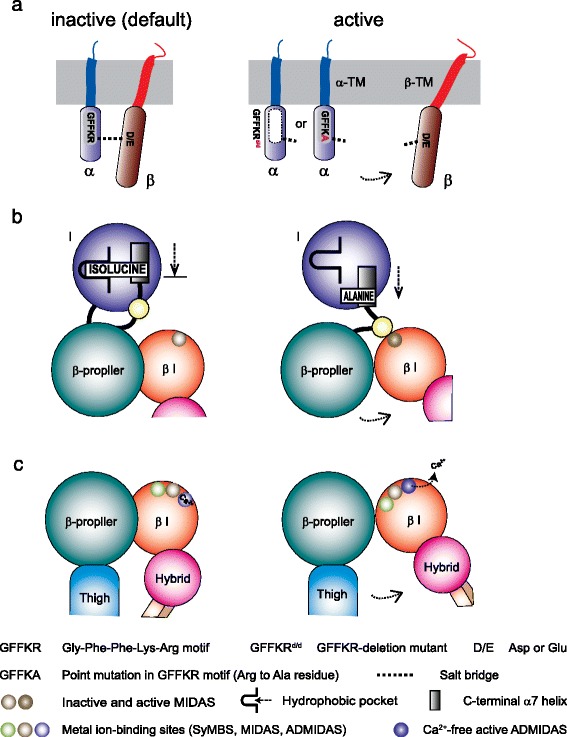
Structural components to stabilize inactive integrin conformation. **a** The cytoplasmic salt bridge is formed between the arginine residue in the α subunit and the glutamate residue in the β subunit, thereby clasping the α/β integrin cytoplasmic tails. A mutation to disrupt the salt bridge results in promoting the cytoplasmic dissociation, thereby inducing integrin activation. **b** A conserved isoleucine at the α I domain C-terminal helix acts as a ratchet to stabilize the inactive conformation. A downward shift of the C-terminal helix is mediated by an interdomain interaction by the β I domain (also known as I-like domain). **c** Ca^2+^ binding site ADMIDAS in the β I domain favors the inactive low-affinity integrin headpiece conformation. A mutation to disrupt the Ca^2+^ binding site induces the constitutively active conformation by facilitating the conformational swing out of the hybrid domain

Integrin molecules contain the intrinsic structural components that favor the default bent conformations, thereby preventing spontaneous aberrant integrin activation in the absence of proper stimulatory signals. The cytoplasmic GFFKR motif constitutes an important intrinsic component that facilitates integrin deactivation, thereby favoring a default inactive conformation. Deletion of the GFFKR motif or mutation of the arginine to alanine are designed to disrupt the cytoplasmic salt bridge, and have been shown to make constitutively active integrins as a result of impaired deactivation (Fig. 3a).

Physiological importance of such integrin deactivation in immune cells has been studied using knock-in mice carrying the mutant αL subunit lacking the GFFKR motif (GFFKR^d/d^; Lfa-1^d/d^) [61]. Lfa-1^d/d^ lymphocytes express the constitutively active αLβ2 that exhibited an impaired integrin deactivation that resulted in persistent cell adhesion but reduced cell migration on ICAM1. When inflammatory response was induced in the peritoneal cavity, Lfa-1^d/d^ mice showed less severe accumulation of neutrophils compared with wild-type mice. Lfa-1^d/d^ T cells showed increased contact time with APCs [2]. However, the increased T cell-APC contact time did not induce productive lymphocyte activation and enhanced proliferation but rather resulted in reduced T-cell proliferation. Thus, appropriate balance of αLβ2 activation and deactivation appears to be important for optimizing T-cell migration and T cell-APC interactions. Notably, in an allograft heart transplantation model, adoptively transferred Lfa-1^d/d^ T cells exhibited reduced capacity to reject the allograft [27].

The physiological importance of the cytoplasmic salt bridge in the integrin α4 subunit was investigated in another study that utilized knock-in mice that carry a specific point mutation in the α4 integrin GFFKR motif [31]. The mutant knock-in mice termed Itga4^GFFKA^ (α4-R974A) showed impaired deactivation of α4β1 and α4β7 integrins [31]. As α4β7 integrin is an important homing receptor to the gut, α4^GFFKA^ mice exhibited a perturbed lymphocyte homing to the gut. On the other hand, naturally occurred mutations in the GFFKR motif of the integrin αIIbβ3 subunit have been reported in the patients suffered with congenital macrothrombocytopenia [32]. The platelet integrin αIIbβ3 in the patients exhibited constitutively active high-affinity state. This could cause aberrant platelet aggregation and consumption, potentially leading to thrombocytopenia.

#### Invariant isoleucine residue

Invariant isoleucine in the α I domain C-terminal helix is another intrinsic component for deactivation. The isoleucine, through a series of hydrophobic interactions (also known as ratcheting interactions), prevents the C-terminal α7-helix from readily moving down, thereby suppressing the conformational conversion to the high-affinity integrin I domain (Fig. 3b). The knock-in (Lfa-1^I306A^ or αL-I306A) mice were generated that carried a specific point mutation (substitution of the isoleucine to arginine) in the integrin αL subunit I domain (Fig. 3b) [52]. αLβ2 deactivation was impaired in the Lfa-1^I306A^ lymphocytes, thereby showing a constitutively active cell adhesion but reduced cell migration on endothelial cells. During migration on ICAM1 substrates, the Lfa-1^I306A^ lymphocytes exhibited an extremely polarized shape characterized with an abnormally prolonged tail. This is because the uropod failed to readily detach from the ligand substrates. Detailed two-photon *in vivo* imaging demonstrated that Lfa-1^I306A^ lymphocytes showed enhanced arrest on the surface of the lymph node endothelial cells, but failed to effectively crawl on and transmigrate across the endothelial cells into tissue parenchyma. This implicates the balance of activation and deactivation in regulating integrin-mediated intravascular crawling that constitutes an integral part of immune-surveillance inside the blood vasculature.

#### ADMIDAS

Unlike the α I domain, the β I domain contains two additional metal binding sites, synergistic metal binding site (SyMBS) and adjacent to MIDAS (ADMIDAS), at the either side of the MIDAS, thereby forming has a linear cluster of three metal ion binding sites (Fig. 3c) [58, 88]. Of note, the ADMIDAS that coordinates Ca^2+^ contributes to stabilizing the C-terminal helix in the low affinity configuration, thereby serving as another intrinsic structural component [6]. The inhibitory effect of Ca^2+^ to integrins is thought to mediate through its coordination to the ADMIDAS of the β I domain. Ca^2+^ coordination at the ADMIDAS in the β7 I domain was disrupted by knocking-in a germline mutation of a functionally key residue (Itgb7^D146A^; β7-D146A) (Fig. 3c) [51]. Itgb7^D146A^ lymphocytes showed the aberrantly activated α4β7 integrin that exhibited perturbed migration on MAdCAM-1 substrates. The Itgb7^D146A^ T cells showed reduced capacity to home to the gut and, thereby, decreased potential to cause intestinal inflammation in a T-cell transfer colitis model. Thus, *in vivo* perturbation of integrin deactivation studied in a series of knock-in mice Lfa-1^d/d^, Lfa-1^I306A^, Itgb7^D146A^, and Itga4^GFFKA^ all points to the anti-migratory and anti-inflammatory phenotypes. This supports the idea that intervention aiming to interfere with not only integrin activation but also integrin deactivation would make an effective therapeutic approach for anti-inflammation.

### Small molecules to modulate integrin activation

A small molecule agonist of αLβ2, compound 4, showed to act as a facilitator of ICAM1 binding by T cells and a simultaneous inhibitor of TEM in a physiological condition [92]. This was due to the finding that compound 4-mediated αLβ2 accumulation in the uropod induced its extreme elongation and the impaired de-adhesion of human lymphocytes. Furthermore, leukadherin, a small molecule agonist of another β2 integrin heterodimer αMβ2, induced the increased αMβ2 dependent cell adhesion of transfectants and of primary human and mouse neutrophils, and the decreased chemotaxis and TEM [41]. Also, TGF-β-related growth differentiation factor-15 (GDF-15) has revealed to be the first cytokine that represses the recruitment of inflammatory leukocytes to inflamed tissues by blocking integrin activation [33].

## Conclusions

The proper balance of activation and deactivation of leukocyte integrins has been shown to be critical for supporting efficient lymphocyte migration and trafficking. We are beginning to understand how the integrin activators (*e.g.*, talin, kindlin) and inactivators (*e.g.*, Dok1, SHARPIN) work together to promote either association or dissociation of the integrin α/β cytoplasmic tails, thereby regulating integrin conformational activation. The intramolecular regulatory sites in integrins are essential for deactivation of integrins. The importance of integrin deactivation in immune cell trafficking to sites of inflammation and activation has been shown in a series of knock-in mice, in which the intramolecular integrin regulatory sites are mutationally disabled. Humanized monoclonal antibodies to leukocyte integrins, natalizumab (α4) and vedolizumab (α4β7), were efficacious in treating inflammatory diseases including Crohn’s disease and ulcerative colitis [15–17, 59, 78, 81]. Small molecule integrin agonists have been reported that interfere with integrin deactivation, thereby suppressing leukocyte extravasation to inflamed tissues. Blocking integrin deactivation might represent a novel approach to alleviate inflammation.
